# Two new species of Ascomycota on bamboo leaves in Fujian, China

**DOI:** 10.3389/fmicb.2025.1555501

**Published:** 2025-06-20

**Authors:** Xiayu Guan, Zhiying Zhao, Nemat O. Keyhani, Taichang Mu, Minhai Zheng, Lixia Yang, Yuchen Mao, Junya Shang, Jiao Yang, Huili Pu, Yongsheng Lin, Mengjia Zhu, Huajun Lv, Zhiang Heng, Huiling Liang, Longfei Fan, Xiaoli Ma, Haixia Ma, Zhenxing Qiu, Junzhi Qiu

**Affiliations:** ^1^Key Laboratory of Ministry of Education for Genetics, Breeding and Multiple Utilization of Crops, College of Horticulture, Fujian Agriculture and Forestry University, Fuzhou, China; ^2^State Key Laboratory of Agricultural and Forestry Biosecurity, College of Life Sciences, Fujian Agriculture and Forestry University, Fuzhou, China; ^3^Department of Biological Sciences, University of Illinois, Chicago, IL, United States; ^4^Guangxi Institute of Botany, Chinese Academy of Sciences, Guilin, China; ^5^College of Plant Protection, Gansu Agricultural University, Lanzhou, China; ^6^College of Life Science and Technology, Xinjiang University, Urumqi, China; ^7^Institute of Tropical Bioscience and Biotechnology, Chinese Academy of Tropical Agricultural Sciences, Hainan Key Laboratory of Tropical Microbe Resources, Haikou, China; ^8^Fuzhou Technology and Business University, Fuzhou, China

**Keywords:** *Didymosphaeriaceae*, *Magnaporthaceae*, fungal phylogenetics, taxonomy, fungal morphology

## Abstract

**Introduction:**

Bamboo is the largest member of the family *Poaceae*, and these fast-growing plants have many beneficial ecological effects. Both mutualistic and pathogenic fungi associate with bamboo, however, only a limited number of such fungal species have been identified, and information concerning the diversity of fungi that parasitizes bamboo leaves is sparse.

**Methods:**

Fungi were isolated from diseased leaves of bamboo from Fujian Province, China. Nucleotide sequences of four genetic loci were used for taxonomic and phylogenetic reconstruction. In addition, the morphological characteristics of fungal structures as well as growth parameters were characterized.

**Results:**

We report two new fungal species, one each within the *Didymosphaeriaceae* and *Magnaporthaceae*, both diverse genera that include saprophytes, endophytes, and pathogens. Based on combined morphological and phylogenetic analyses, including the nucleotide sequences of the internal transcribed spacer (ITS), the 28S large subunit of ribosomal RNA (LSU), the 18S small subunit of nuclear ribosomal RNA gene (SSU), the large subunit of RNA polymerase I (*rpb1*), and the translation elongation factor 1-α gene (*tef1-*α), the two new species described herein have been named as *Bifusisporella magnum* sp. nov., and *Letendraea bambusae* sp. nov. Detailed descriptions and morphological features of the species are provided.

**Discussion:**

These data identify and describe new species within the *Didymosphaeriaceae* and *Magnaporthaceae*, expanding the diversity and distribution of fungi associating with bamboo.

## 1 Introduction

Bamboo (*Bambusoideae, Poales*) is one of the most important forms of wood in the world (Wysocki et al., 2015), with rich species diversity. It is found in tropical, subtropical, and temperate regions on several continents, albeit rare in Europe (Phookamsak et al., 2022). Global bamboo resources are abundant, with a total of 140 genera and over 1,750 species. In China, there are ~770 species of bamboo, along with five hybrids and 203 forms or varieties, spread across 42 genera (Yang et al., 2024). China boasts the richest bamboo resources, the highest diversity, and the largest total cultivation area, accounting for more than 50% of the world's bamboo species (Liu et al., 2018; Dlamini et al., 2022). Bamboo species are widely distributed from cold mountain regions to hot tropical regions, form beneficial associations with a variety of fungi, but can also be vulnerable to fungal infection. Plant-associated fungi can be endophytic, living within plant tissues, exist on the surface of the plant as epiphytes, function as saprobes, and/or form parasitic interactions, leading to plant disease (Mu et al., 2024). Currently, more than 1,100 species of fungi associated with bamboo have been documented or described globally (Dai et al., 2018; Jiang et al., 2022). Intriguingly, several “bambusicolous” fungi have been used in Chinese medicine to treat various human diseases and are known to produce a variety of bioactive functional metabolites (Yan et al., 2023).

Studies of bambusicolous fungi have been carried out since the 18th century, the earliest of which was initiated by Léveillé (1845), and aspects of their molecular phylogeny developed by Tanaka et al. (2009), who established a new family, *Tetraplosphaeriaceae*, and assigned it within the order *Pleosporales*. A second, diverse family of bamboo associating fungi, *Didymosphaeriaceae*, also within *Pleosporales*, has further been defined (Ariyawansa et al., 2014b; Dai et al., 2016). Species within these families are widely distributed globally, can grow on and/or parasitize a variety of hosts, including plants and occasionally other fungi and animals including human. These families encompass endophytes, epiphytes, saprophytes, and pathogens typically found on woody branches, herb stems, leaves, pods, and soil (Ren et al., 2022).

Munk (1953) introduced *Didymosphaeriaceae* in the order *Melanommatales*, with the type genus *Didymosphaeria* Fuckel (Barr, 1990). The sexual morph of these fungi is characterized by one-septal ascospores and trabeculate pseudoparaphyses which anastomose mostly above the asci (Ariyawansa et al., 2014a). The asexual morphs of *Didymosphaeriaceae* are diverse (Wijayawardene et al., 2022). Members of the genus of *Montagnulaceae* display one to multi-septate ascospores and generally cellular pseudoparaphyses. However, Ariyawansa et al. (2014a) compared the new species *Didymosphaeria rubi-ulmifolii* with the type species, *D. futilis* (Berk. & Broome) Rehm, based on multilocus phylogenetic analyses, demonstrating that *D. rubi-ulmifolii* clustered as a separate genus in *Montagnulaceae*, indicating that *Montagnulaceae* and *Didymosphaeriaceae* are synonymous (Ariyawansa et al., 2014b). *Letendraea* belongs to the *Didymosphaeriaceae* of the *Pleosporales*. Some species in the genus *Letendraea*, such as *Letendraea* sp. WZ07, are considered endophytic fungi that can colonize healthy plant tissues without causing obvious disease symptoms, and these fungi have been shown to produce secondary metabolites with anti-inflammatory activity (Qiao et al., 2022; Yan et al., 2024).

The order *Magnaporthales*, belongs to the class *Sordariomycetes* within the phylum Ascomycota. Wijayawardene et al. (2022) proposed five families and 39 genera within this order. *Magnaporthaceae* is the most diverse family within the *Magnaporthales* (Feng et al., 2024). Cannon (1994) proposed the *Magnaporthaceae* family to accommodate *Magnaporthe* and its related genus *Buergenerula*. As early as 2009, *Magnaporthaceae* was independently classified as a new order, *Magnaporthales*, based on morphological characteristics and phylogenetic analysis of LSU and SSU genes (Thongkantha et al., 2009). Asexual forms appear as simple, unbranched or branched conidia, conidia straight or curved, transparent to light brown, septate, conidiogenous cells have pigment (Klaubauf et al., 2014). Members of the *Magnaporthaceae* include important plant pathogens of rice, grasses, and cereals. During an investigation of endophytic fungi in Brazilian *Sorghum bicolor* leaves, two isolates were discovered that exhibited morphological and phylogenetic differences from previously known members within the *Magnaporthaceae*. Consequently, Silva et al. (2019) established a new genus, *Bifusisporella* to accommodate these isolates.

In this study, we report on the identification and characterization of two species isolated from diseased bamboo plants, namely: *Bifusisporella magnum* sp. nov. (*Magnaporthaceae*) and *Letendraea bambusae* sp. nov. (*Didymosphaeriaceae*), based on phylogenetic (molecular) and morphological analyses. Phylogenetic reconstruction involved a multi-locus approach, combining data from five genetic loci: the intervening 5.8S nrRNA gene (ITS), the 28S large subunit of nuclear ribosomal RNA gene (LSU), the 18S small subunit of nuclear ribosomal RNA gene (SSU), the large subunit of RNA polymerase I (*rpb1*), and the translation elongation factor 1-α gene (*tef1-*α) nucleotide sequences. These results expand the diversity of fungi infecting bamboo plants and provide distinguishing characteristics of bambusicolous fungi to better elucidate relationships between species.

## 2 Materials and methods

### 2.1 Sampling and isolation

Fungal specimens isolated from diseased leaves of bamboo were collected in Fujian Province, China. Diseased leaves with obvious fungal necrosis and/or typical blight-spot symptoms were cut into ~25 mm^2^ pieces. The fragments were soaked in 75% ethanol solution for 60 s, washed once, then soaked in sterile deionized water for 20 s, then transferred to 5% sodium hypochlorite for 30–40 s, and then washed in sterile deionized water for three times for 45–60 s. The washed fragments were placed on sterilized filter paper to dry, then transferred to PDA plates and incubated at 25°C for 7 days (Su et al., 2021). Fungal hyphae on the growing edges of colonies were transferred to a new PDA plate until pure fungal cultures were obtained. Spore production and visual appearance of the colonies were examined on SNA plates amended with sterilized pine needles or bamboo leaf fragments, and plates were incubated at 25°C under a regimen of 12 h of near-ultraviolet light and 12 h of darkness (Zhang et al., 2023).

### 2.2 Morphological observation

After 7–14 days of incubation, fungal colonies were photographed with a digital camera (Canon EOS 6D MarkII, Tokyo, Japan) to detail the morphology of the conidiomata, conidiophores, and conidiogenous cells using a stereomicroscope (Nikon SMZ745, Tokyo, Japan) and biological microscope (Ni-U, Tokyo, Japan) with a digital camera (Olympus, Tokyo, Japan). Images were analyzed using Digimizer 5.4.4 software for various size parameters of fungal structures. A single colony derived from the purified culture was cut into 1 cm^2^ pieces and preserved in 10% glycerol and sterile water at 4°C for further detailed study.

### 2.3 DNA extraction, PCR amplification and sequencing

A fungal DNA Mini-kit (OMEGA-D3390, Feiyang Biological Engineering Corporation, Guangzhou, China) was used to extract fungal genomic DNA from growing hyphae. Genetic loci corresponding to the ITS region were amplified by polymerase chain reaction (PCR) using primers ITS5 and ITS4 (White et al., 1990; Zou et al., 2022), with primers LR0R and LR5 used to amply the LSU region (Vilgalys and Hester, 1990), primers NS1 and NS4 for the SSU region (White et al., 1990), and primers fRPB1-Ac and fRPB1-Cr for amplification of the *rpb1* gene (Matheny et al., 2002; Castlebury et al., 2004), The *tef1-*α locus was amplified using EF1-983F and EF1-2218R (Rehner and Buckley, 2005). Primer sequences are given in Table 1.

**Table 1:** Gene regions and PCR primers and programs used in this study.

**Gene**	**Primers**	**Sequence (5^′^-3^′^)**	**PCR cycles**	**References**
ITS	ITS5	GGA AGT AAA AGT CGT AAC AAG G	(95°C: 30 s, 55°C: 30 s, 72°C: 1 min) × 35 cycles	White et al., 1990
	ITS4	TCC TCC GCT TAT TGA TAT GC		
LSU	LR0R	GTA CCC GCT GAA CTT AAG C	(95°C: 30 s, 52°C: 30 s, 72°C: 1 min) × 35 cycles	Vilgalys and Hester, 1990
	LR5	TCC TGA GGG AAA CTT CG		
SSU	NS1	GTA GTC ATA TGC TTG TCT C	(95°C: 30 s, 50°C: 30 s, 72°C: 1 min) × 35 cycles	White et al., 1990
	NS4	CTT CCG TCA ATT CCT TTA AG		
*rpb1*	fRPB1-Ac	GAR TGY CCD GGD CAY TTY GG	(95°C: 30 s, from 57°C to 72°C at 0.2°C/s:30 s, 72°C: 1 min) × 35 cycles	Matheny et al., 2002; Castlebury et al., 2004
	fRPB1-Cr	CCNGCDATNTCRTTRTCCATRTA		
*tef1-α*	EF1-983F	GCYCCYGGHCAYCGTGAYTT	(95°C: 30 s, 57°C: 30 s, 72°C: 1 min) × 35 cycles	Rehner and Buckley, 2005
	EF1-2218R	ATGACACCRACRGCRACRGTYTGYAT		

PCR amplification was performed using a Bio-Rad thermal cycler (Hercules, CA, USA). Amplification reactions were performed in 25 μl reaction volumes consisting of 12.5 μl 2 × Rapid Taq Master Mix (Vazyme, Nanjing, China), 1 μl forward and reverse primers (10 μM) (Sangon, Shanghai, China), and 1 μl template genomic DNA, adjusted with sterile deionized water to a total of 25 μl. The integrity and sizes of PCR products were estimated by 1% agarose gel electrophoresis. All PCR products were purified, and their nucleotide sequences were determined by a commercial service (Tsingke Company Limited, Fuzhou, China). Sequence assembly was performed using MEGA 7.0 (Kumar et al., 2016). The new sequences generated in this study have been uploaded to GenBank (https://www.ncbi.nlm.nih.gov, accession numbers given in Table 2).

**Table 2:** Species and GenBank accession numbers of DNA sequences used in this study, with new sequences indicated in bold.

**Species**	**Culture/voucher**	**Country**	**GenBank accession number**				
			**ITS**	**LSU**	**SSU**	***tef1-**α*	***rpb1***
*Barretomyces calatheae*	CBS 129274 = CPC 18464	Brazil	KM484831	KM484950	–	–	KM485045
*Bambusicularia brunnea*	CBS 133599	Japan	KM484830	KM484948	–	–	KM485043
*Bambusicularia brunnea*	CBS 133600	Japan	AB274436	KM484949	–	–	KM485044
*Bifusisporella bambooensis*	CGMCC3.25653	China	PP159031	PP159039	–	PP488459	PP488463
*Bifusisporella bambooensis*	CGMCC3.27207	China	PP477445	PP477439	–	PP488461	PP488465
*Bifusisporella fujianensis*	CGMCC3.25651	China	PP159030	PP159038	–	PP488458	PP488462
*Bifusisporella fujianensis*	CGMCC3.27206	China	PP477444	PP477438	–	PP488460	PP488464
*Bifusisporella fusispora*	SICAU 23-0027	China	OR405944	OR405925	–	OR947488	–
*Bifusisporella graminicola*	YNE00934	China	MW479074	MW478089	–	MW482636	MW482801
*Bifusisporella graminicola*	YNE01000	China	MW479075	MW478090	–	MW482641	MW482802
*Bifusisporella graminis*	YNE01143	China	MW479087	MW478068	–	MW482644	MW482809
*Bifusisporella graminis*	YNE00994	China	MW479084	MW478066	–	MW482643	MW482808
***Bifusisporella magnum*** **sp. nov**.	**CGMCC3.27816** ^**T**^	**China**	**PQ380230**	**PQ380232**	**–**	**PQ45631**	**PQ456329**
***Bifusisporella magnum*** **sp. nov**.	**CGMCC3.27817**	**China**	**PQ380231**	**PQ380233**	**–**	**PQ45632**	**PQ45630**
*Bifusisporella sorghi*	URM 7442	Brazil	MK060155	MK060153	–	MK060157	MK060159
*Bifusisporella sorghi*	URM 7864	Brazil	MK060156	MK060154	–	MK060158	MK060160
*Bifusisporella sichuanensis*	SICAUCC 22-0073	China	ON227097	ON227101	–	ON244427	ON244428
*Bussabanomyces longisporus*	CBS 125232	Thailand	KM484832	KM484951	–	KM009202	KM485046
*Buergenerula spartinae*	ATCC 22848	USA	JX134666	DQ341492	–	JX134692	JX134720
*Falciphora oryzae*	CBS 125863 = R 5-6-l	China	EU636699	KJ026705	–	JN857963	KJ026706
*Falciphoriella solaniterrestris*	CBS 117.83	Netherlands	KM484842	KM484959	–	–	KM485058
*Gaeumannomycella graminis*	CPC 26020 = CBS 141384	USA	KX306498	KX306568	–	KX306701	KX306633
*Gaeumannomycella graminicola*	CPC 26025 = CBS 141381	USA	KX306495	KX306565	–	KX306698	KX306630
*Gaeumannomycella caricis*	CPC 26262 = CBS 141374	UK	KX306478	KX306548	–	KX306675	KX306671
*Gaeumannomycella caricis*	CBS 388.81	UK	KM484843	KM484960	–	KX306674	–
*Gaeumannomyces floridanus*	CPC 26037	USA	KX306491	KX306561	–	KX306693	KX306626
*Gaeumannomyces fusiformis*	CPC 26068	USA	KX306492	KX306562	–	KX306694	KX306627
*Gaeumannomyces glycinicola*	CPC 26266	USA	KX306494	KX306564	–	KX306696	KX306629
*Gaeumannomyces glycinicola*	CPC 26057	USA	KX306493	KX306563	–	KX306695	KX306628
*Gaeumannomyces graminicola*	CBS 352.93	–	KM484834	DQ341496	–	KX306697	KM485050
*Gaeumannomyces graminis*	CPC 26045	–	KX306505	KX306575	–	KX306708	KX306640
*Gaeumannomyces graminis* var. *graminis*	M33	–	JF710374	JF414896	–	JF710411	JF710442
*Gaeumannomyces graminis* var. *graminis*	M54	–	JF414848	JF414898	–	JF710419	JF710444
*Gaeumannomyces hyphopodioides*	CBS 350.77	UK	KX306506	KX306576	–	–	–
*Gaeumannomyces hyphopodioides*	CBS 541.86	Germany	KX306507	KX306577	–	KX306709	–
*Gaeumannomyces oryzicola*	CPC 26063	USA	KX306516	KX306586	–	KX306717	KX306646
*Gaeumannomyces oryzinus*	CPC 26030	Bahamas	KX306517	KX306587	–	KX306718	KX306647
*Gaeumannomyces radicicola*	CBS 296.53	Canada	KM009170	KM009158	–	KM009206	KM009194
*Gaeumannomyces setariicola*	CPC 26059	South Africa	KX306524	KX306594	–	KX306725	KX306654
*Gaeumannomyces tritici*	CBS 273.36	Argentina	KX306525	KX306595	–	KX306729	KX306655
*Gaeumannomyces walkeri*	CPC 26028	USA	KX306543	KX306613	–	KX306746	KX306670
*Gaeumannomyces wongoonoo*	BRIP:60376	Australia	KP162137	KP162146	–	–	–
*Kohlmeyeriopsis medullaris*	CBS 117849 = JK5528S	USA	KM484852	KM484968	–	–	KM485068
*Macgarvieomyces borealis*	CBS 461.65	UK	MH858669	DQ341511	–	KM009198	KM485070
*Macgarvieomyces juncicola*	CBS 610.82	Netherlands	KM484855	KM484970	–	KM009201	KM485071
*Magnaporthaceae incertaesedis*	CPC 26284 = GP57	UK	KX306546	KX306616	–	KX306677	–
*Magnaporthiopsis* sp.	CPC 26038	USA	KX306545	–	–	KX306676	KX306672
*Magnaporthiopsis incrustans*	M35	–	JF414843	JF414892	–	–	JF710437
*Magnaporthiopsis maydis*	CBS 133165	Israel	KX306544	KX306614	–	–	–
*Magnaporthiopsis maydis*	CBS 662.82A	Egypt	KM484856	KM484971	–	–	KM485072
*Magnaporthiopsis cynodontis*	RS7-2 = CBS 141700	USA	KJ855508	KM401648	–	KP282714	KP268930
*Magnaporthiopsis cynodontis*	RS5-5	USA	KJ855506	KM401646	–	KP282712	KP268928
*Magnaporthiopsis cynodontis*	RS3-1	USA	KJ855505	KM401645	–	KP282711	KP268927
*Magnaporthiopsis meyeri-festucae*	FF2	–	MF178146	MF178151	–	MF178167	MF178162
*Magnaporthiopsis meyeri-festucae*	SCR11	–	MF178150	MF178155	–	MF178171	MF178166
*Magnaporthiopsis panicorum*	CM2S8	–	KF689643	KF689633	–	KF689623	KF689613
*Magnaporthiopsis panicorum*	CM10s2	–	KF689644	KF689634	–	KF689624	KF689614
*Magnaporthiopsis rhizophila*	M22	–	JF414833	JF414882	–	JF710407	JF710431
*Nakataea oryzae*	CBS 332.53	USA	KM484867	KM484981	–	–	KM485083
*Nakataea oryzae*	CBS 252.34	Burma	KM484862	KM484976	–	–	KM485078
*Nakataea oryzae*	CBS 288.52	Japan	KM484864	KM484978	–	–	KM485080
*Neogaeumannomyces bambusicola*	MFLUCC 11-0390	Thailand	KP744449	KP744492	–	–	–
*Neopyricularia commelinicola*	CBS 128307 = KACC 44083	Korea	FJ850125	KM484984	–	KM009199	KM485086
*Neopyricularia commelinicola*	CBS 128308	Korea	FJ850122	KM484985	–	–	KM485087
*Omnidemptus affinis*	ATCC 200212	Australia	JX134674	KX134686	–	JX134700	JX134728
*Omnidemptus affinis*	YNE00716	–	MW478951	–	–	MW482673	MW482812
*Omnidemptus affinis*	YNE00622	–	MW478950	MW478069	–	MW482672	MW482811
*Omnidemptus graminis*	CBS 138107	–	MK487758	MK487734	–	MK495980	–
*Omnidemptus graminis*	ZJE01803	–	MW478956	–	–	–	–
*Ophioceras dolichostomum*	CBS 114926 = HKUCC 3936 = KM 8	China	JX134677	JX134689	–	JX134703	JX134731
*Ophioceras leptosporum*	CBS 894.70 = ATCC 24161 = HME 2955	UK	JX134678	JX134690	–	JX134704	JX134732
*Proxipyricularia zingiberis*	CBS 132355	Japan	AB274433	KM484987	–	–	KM485090
*Proxipyricularia zingiberis*	CBS 133594	Japan	AB274434	KM484988	–	–	KM485091
*Pseudophialophora eragrostis*	CM12m9	USA	KF689648	KF689638	–	KF689628	KF689618
*Pseudopyricularia cyperi*	CBS 133595	Japan	KM484872	KM484990	–	–	AB818013
*Pseudopyricularia kyllingae*	CBS 133597	Japan	KM484876	KM484992	–	KT950880	KM485096
*Pyricularia grisea*	BR0029	Brazil	KM484880	KM484995	–	–	KM485100
*Pyricularia grisea*	CR0024	Korea	KM484882	KM484997	–	–	KM485102
*Pyricularia ctenantheicola*	GR0001 = Ct-4 = ATCC 200218	Greece	KM484878	KM484994	–	–	KM485098
*Pyricularia oryzae*	CBS 365.52 = MUCL 9451	Japan	KM484890	KM485000	–	–	KM485110
*Slopeiomyces cylindrosporus*	CBS 609.75	UK	KM484944	KM485040	–	JX134693	KM485158
*Utrechtiana cibiessia*	CBS 128780 = CPC 18916	Netherlands	JF951153	JF951176	–	–	KM485047
*Xenopyricularia zizaniicola*	CBS 132356	Japan	KM484946	KM485042	–	KM009203	KM485160
*Kalmusia araucariae*	CPC 37475	USA	MT223805	MT223900	–	–	–
*Kalmusia cordylines*	ZHKUCC 21-0092	China	NR_184482	NG_088312	–	–	–
*Kalmusia cordylines*	ZHKU 21-0003	China	–	OL818333	OL818335	–	–
*Kalmusia ebuli*	CBS 123120	–	–	JN644073	JN851818	–	–
*Kalmusia erioi*	MFLU 18-0832	Thailand	–	MN473052	–	MN481599	–
*Kalmusia spartii*	MFLUCC 14-0560	–	–	NG_070926	NG_063569	–	–
*Kalmusia italica*	MFLUCC 13-0066	Thailand	–	NG_070925	NG_070109	–	–
*Kalmusia longispora*	T15142	–	MN945157	MN945151	–	–	–
*Kalmusia longispora*	CBS:582.83	Canada	–	MH873371	–	–	–
*Kalmusia longispora*	CBS:824.84	Germany	MH861838	MH873526	–	–	–
*Kalmusia sarothamni*	CBS 116474	China	KF796676	KF796673	KF796672	–	–
*Kalmusia sarothamni*	CBS 113833	China	–	KF796671	KF796670	–	–
*Kalmusia spartii*	MFLU 14-0751	Thailand	KP744441	KP744487	KP753953	–	–
*Kalmusia utahensis*	CBS:127794	USA	MH864709	MH876142	–	–	–
*Kalmusia variispora*	CBS 121517	Syria	MH863113	MH874668	–	–	–
*Laburnicola hawksworthii*	MFLUCC 13-0602	Thailand	KU743194	KU743195	KU743196	–	–
*Laburnicola muriformis*	MFLUCC 16-0290	Thailand	KU743197	KU743198	KU743199	KU743213	–
*Laburnicola muriformis*	MFLUCC 14-0921	Thailand	KU743200	KU743201	KU743202	–	–
*Letendraea magnoliae*	MFLUCC 19-0052	Thailand	MW222160	–	–	MW233021	–
***Letendraea bambusae*** **sp. nov**.	**CGMCC3.28309** ^**T**^	**China**	**PQ218951**	**PQ218949**	**PQ218947**	**PQ282134**	–
***Letendraea bambusae*** **sp. nov**.	**CGMCC3.28310**	**China**	**PQ218952**	**PQ218950**	**PQ218948**	**PQ282135**	–
*Letendraea cordylinicola*	MFLUCC 11_0148	Thailand	NR_154118	NG_059530	NG_068362	–	–
*Letendraea cordylinicola*	MFLUCC11_0150	Thailand	KM213996	KM213999	KM214001	–	–
*Letendraea helminthicola*	MFLUCC:19-0055	–	MW222161	MW209732	MW209735	MW233020	–
*Letendraea helminthicola*	CBS884_85	China	MK404145	AY016362	AY016345	MK404174	–
*Letendraea padouk*	CBS485.70	China	–	AY849951	–	–	–
*Letendraea eurotioides*	CBS 212.31	–	–	AY787935	–	–	–
*Letendraea* sp.	UTHSC:DI16-267	–	LT796861	LN907410	–	LT797101	–
*Letendraea* sp.	UTHSC:DI16-370	–	LT796908	LN907513	–	LT797148	–
*Letendraea* sp.	UTHSC:DI16-351	–	LT796897	LN907494	–	LT797137	–
*Spegazzinia tessarthra*	SH 287	Japan	–	AB807584	AB797294	AB808560	–
*Spegazzinia radermacherae*	C264	Thailand	MK347740	MK347957	MK347848	–	–

Ex-type strains are labeled with “T” in this study.

### 2.4 Sequence alignment and phylogenetic analysis

Sets of orthologous sequences for all loci were downloaded from GenBank. All sequences were aligned using the MAFFT v. 7 online service (http://mafft.cbrc.jp/alignment/server/, accessed on 27 September 2024) (Katoh et al., 2019) and manually adjusted in BioEdit v .7.2.6.1 (He et al., 2022) and MEGA 7.0 (Kumar et al., 2016). Using Maximum Likelihood (ML) and Bayesian Inference (BI) analyses, phylogenetic trees were constructed to explore the evolutionary relationships of fungi within the *Magnaporthaceae* and *Didymosphaeriaceae* families, respectively. *Spegazzinia tessarthra* (SH 287) and *Spegazzinia radermacherae* (C264) were selected as outgroup taxa of *Didymosphaeriaceae*. *Ophioceras dolichostomum* (CBS 114926) and *Ophioceras leptosporum* (CBS 894.70) (*Ophioceraceae, Magnaporthales*) were used as outgroup taxa of *Magnaporthaceae* (Zhao et al., 2024).

Bayesian Posterior Probabilities (BPP) was assessed using Markov Chain Monte Carlo (MCMC) methods (Rannala and Yang, 1996; Zhaxybayeva and Gogarten, 2002). Two parallel runs were conducted, each with four chains, starting from 2,000,000 generations of random trees, sampling every 100 generations, resulting in a total of 20,000 trees, the average standard deviation of split frequencies was set to be < 0.01, starting from random trees (Ronquist and Huelsenbeck, 2003). The first 25% of the trees were discarded as burn-in, and the remaining 75% were used to calculate the Bayesian Posterior Probabilities (BPP) for the majority rule consensus tree (Ji et al., 2022). The consensus trees were visualized in FigTree v.1.4.4 (https://tree.bio.ed.ac.uk/software/figtree; Rambaut, 2018) and were embellished using Adobe Illustrator CS 6.0 (Adobe Systems Inc., USA). Maximum likelihood bootstrap support values (≥70%) and Bayesian Posterior Probabilities (≥0.90) were considered as significantly supported branches. The new sequences generated in this study have been deposited in GenBank (https://www.ncbi.nlm.nih.gov; accessed on 12 October 2024).

## 3 Results

### 3.1 Phylogenetic analysis

A total of two samples were collected from diseased bamboo leaves. Of these, four purified fungal colonies were obtained. Genomic DNA was extracted from all specimens and the nucleotide sequences of the internal transcribed spacer (ITS), the 28S large subunit of ribosomal RNA (LSU), the 18S small subunit of nuclear ribosomal RNA gene (SSU), the large subunit of RNA polymerase I (*rpb1*), the translation elongation factor 1-α gene (*tef1-*α) gene were determined as detailed in the Materials and Methods section.

**Figure 1:**
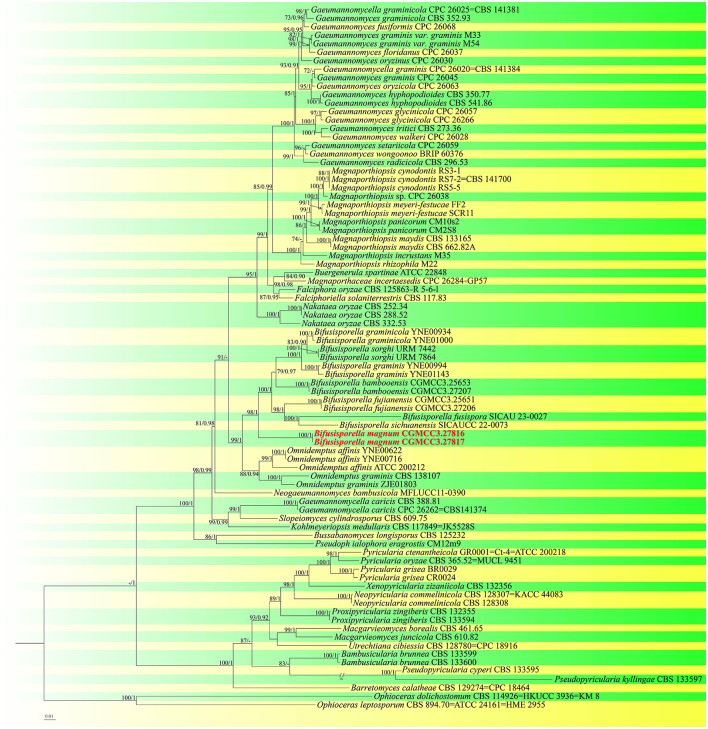
Molecular phylogenetic analysis of *Magnaporthaceae* and *Pyriculariaceae* based on a combined ITS, LSU, *tef1-*α, and *rpb1* sequences. IQ-TREE ultra-fast bootstrap (BS) (UFBoot) values ≥70% and MrBayes posterior probability (PP) ≥0.90 are displayed above or below the respective branches (ML/BI). The newly described species is marked red.

For construction of the phylogenetic tree of isolates matching *Magnaporthaceae*, the concatenated sequence dataset of ITS, LSU, *tef1-*α, and *rpb1* was used in analyses which included 83 taxa with *Ophioceras dolichostomum* (CBS 114926) and *Ophioceras leptosporum* (CBS 894.70) as the outgroup (Figure 1). For each fungal isolate, 2,869 bp of nucleotide sequences corresponding to portions of the ITS, LSU, *rpb1*, and *tef1-*α loci (ITS: 1–433; LSU: 434–1,213; *rpb1*: 1,214–1,981; *tef1-*α: 1,982–2,869) were isolated. Based on these and morphological data, a new species, *Bifusisporella magnum*, was identified, related to *Bifusisporella bambooensis* (CGMCC3.25653), with high support (ML-BS: 98% and BYPP: 1). For *Bifusisporella magnum*, 1,346 distinct and 398 variable nucleotide sequences, including gaps were identified, with 909 patterns parsimony informative. ModelFinder (Kalyaanamoorthy et al., 2017) was used to select the best-fit model using AIC criterion, maximum likelihood phylogenies were inferred using IQ-TREE (Nguyen et al., 2015) under the GTR+R4+F model for 5000 ultrafast (Minh et al., 2013) bootstraps, as well as the Shimodaira–Hasegawa–like approximate likelihood-ratio test (Guindon et al., 2010) (Lsetnst = 1, rates = Invgamma). Average standard deviation of split frequencies was 0.004924. Based on these analyses, the two isolates of *Bifusisporella* described herein represent one novel species.

**Figure 2:**
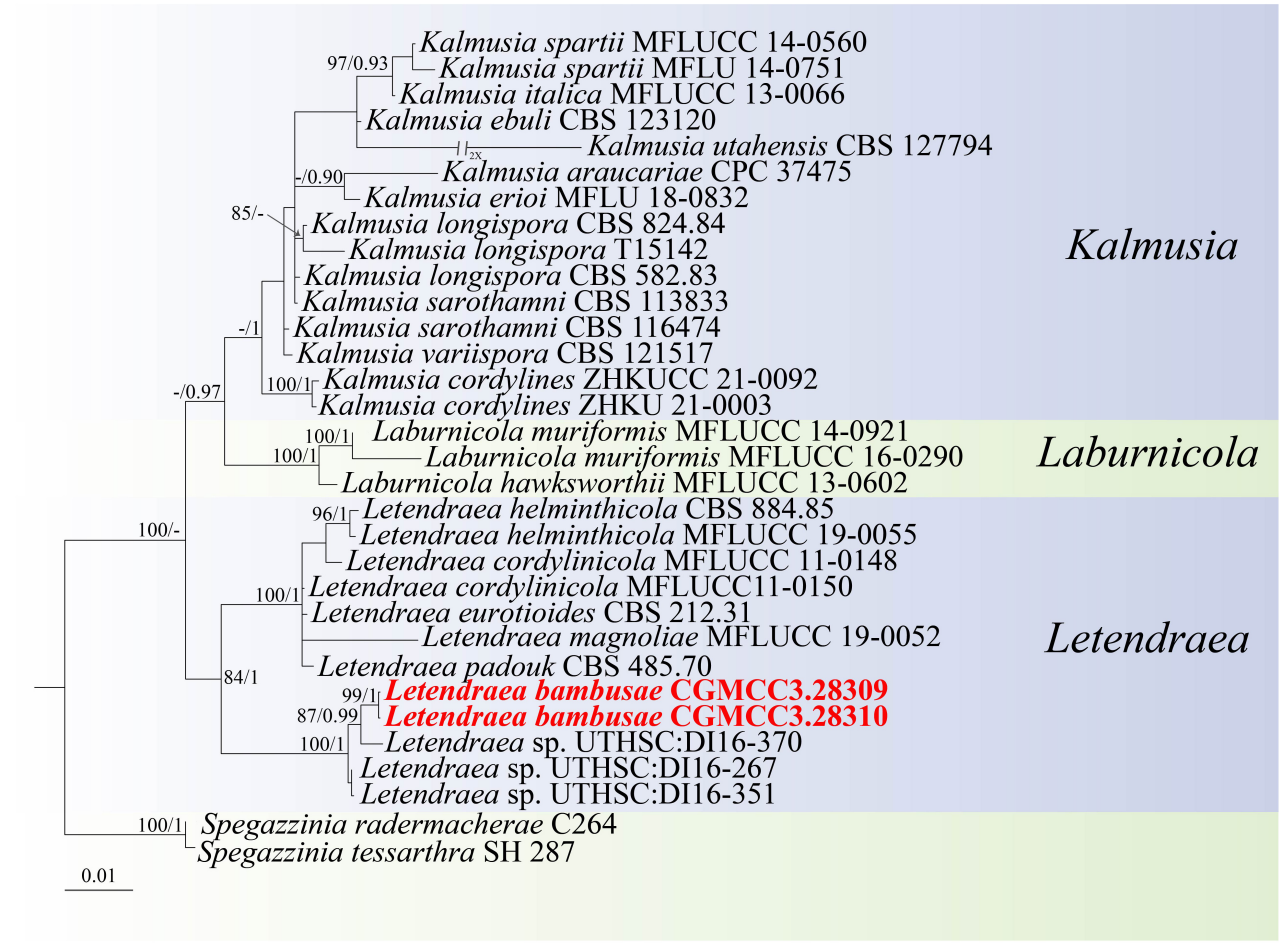
Phylogenetic tree of *Didymosphaeriaceae* inferred from Bayesian inference analyses base on a combined ITS, LSU, SSU, and *tef1-*α sequence dataset, with *Spegazzinia tessarthra* (SH 287) and *Spegazzinia radermacherae* (C264) as outgroup. IQ-TREE ultra-fast bootstrap (BS; UFBoot) values ≥70% and MrBayes posterior probability (PP) ≥0.90 are displayed above or below the respective branches (ML/BI). The newly described species is marked in red.

For the *Letendraea* isolates, in our dataset, the ITS + LSU + SSU + *tef1-*α concatenated sequence dataset had an aligned length of 2,654 total characters (ITS: 1–405, LSU: 406–1,176, SSU: 1,177–1,941, *tef1-*α: 1,942–2,654) including gaps, of which 2,282 characters were constant, 151 were variable and parsimony-uninformative, and 221 bp were parsimony-informative. ModelFinder (Kalyaanamoorthy et al., 2017) was employed to choose the best-fit model based on AIC criterion, maximum likelihood phylogenies were inferred using IQ-TREE (Nguyen et al., 2015) under the TIM2e+R2 model for 5,000 ultrafast (Minh et al., 2013) bootstraps, as well as the Shimodaira–Hasegawa–like approximate likelihood-ratio test (Lsetnst = 1, rates = Equal) (Guindon et al., 2010). Bayesian analyses resulted in an average standard deviation of split frequencies = 0.003168. The topology shown by ML and BI were almost identical, although there were slight differences in statistical support for some branches (Figure 2). Based on phylogenetic resolution and morphological analysis, we report a new species of *Didymosphaeriaceae*: *Letendraea bambusae*, with the two strains studied herein representing one novel species. The new species *Letendraea bambusae* clustered alongside *Letendraea* sp. (UTHSC: DI16-267) and formed a clade sister to *Letendraea* sp. (UTHSC: DI16-267) with a posterior probability of 1.

### 3.2. Taxonomy

**Figure 3:**
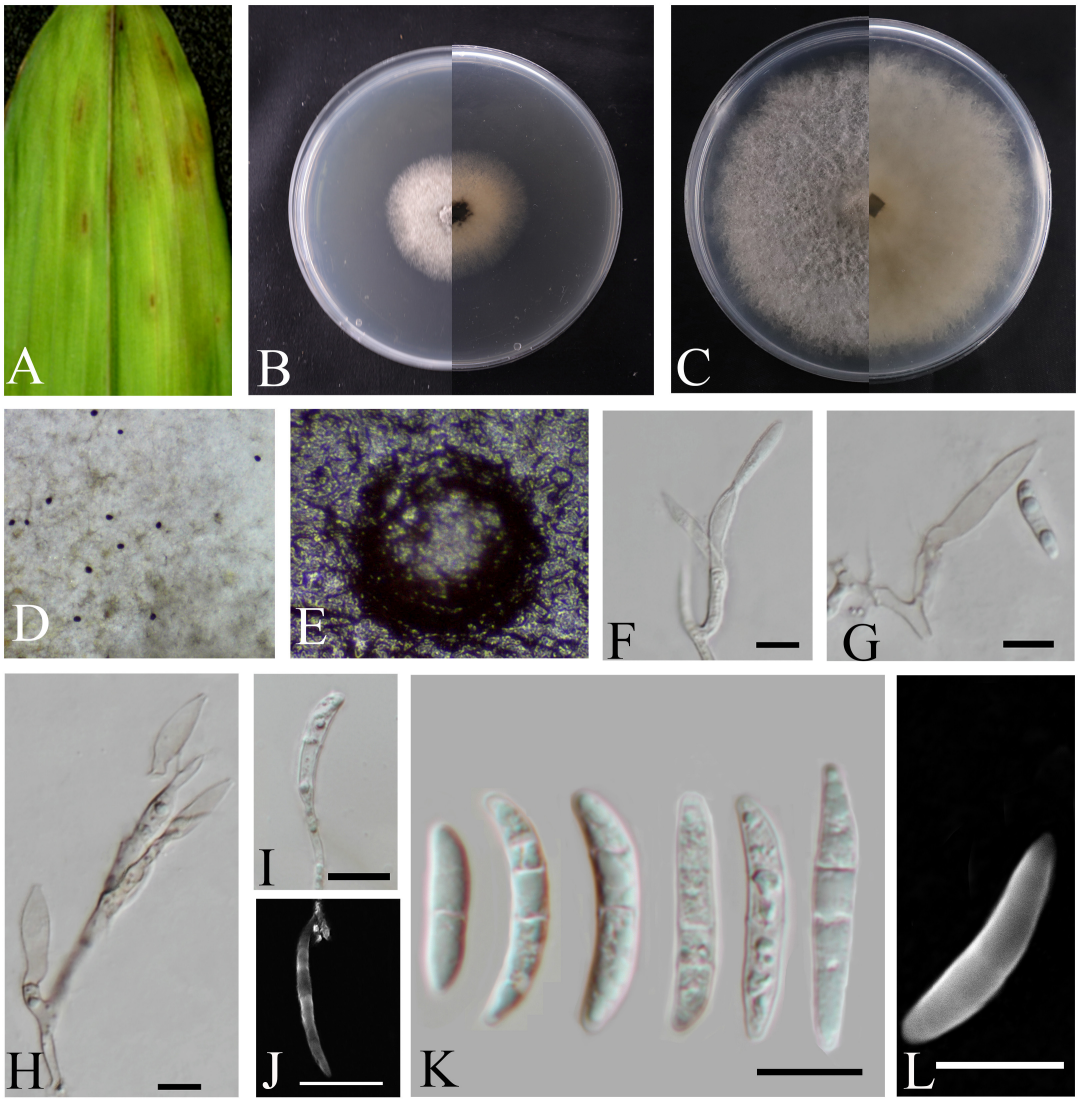
*Bifusisporella magnum* (HMAS353209). **(A)** Leaves of host plant. **(B, C)** Upper and reverse view of colony after incubation for 7 days on PDA and 14 days. **(D, E)** Conidiomata sporulating on PDA. **(F–J)** Conidiogenous cells and conidia. **(K, L)** Conidia. **(F–L)** Scale bars: 10 μm.

***Bifusisporella magnum*** sp. nov. Z.Y. Zhao, X.Y. Guan and J.Z. Qiu (Figure 3).

MycoBank: MB856574

Holotype: China, Fujian Province, Fujian Agriculture and Forestry University (119°14′35.14″E, 26°5′2.55″N), from diseased leaves of bamboo in China, March 2023, Z.Y. Zhao (holotype HMAS353209; ex-type living culture CGMCC3.27816).

Etymology: From Latin magnus (“great”). In reference to the large sized conidiogenous cells.

Description: The oval-shaped leaf spots feature a gradient of color, shifting from white at the periphery to brown at the center. *Conidiomata* emits droplets that form distinct, black, spherical protrusions on the agar surface. *Conidiophores* reduced to *conidiophores cells. Conidiogenous cells* are cylindrical or pot-shaped, solitary or clustered, curved, and elongated in shape. 18.8–30.4 × 4.6–6.4 μm, mean ± SD = 22.5 ± 4.7 × 5.4 ± 0.9 μm, *n* = 20. *Conidia* are sickle-shaped or crescent-shaped, with a smooth or cracked surface, and are transparent. 0–4 septa, 11.8–28.9 × 2.1–4.3 μm, mean ± SD = 21.2 ± 4.4 × 3.5 ± 0.6 μm, n = 30.

Culture characteristics: Colonies on PDA are fluffy, velvety, and grayish-white, calculated growth rate of 0.6 cm/day at 25°C.

Material examined: China, Fujian Province, Fujian Agriculture and Forestry University (119°14′35.14″E, 26°5′2.55″N), from diseased leaves of bamboo in China, March 2023, Z.Y. Zhao (paratype HMAS353210; ex-paratype living culture CGMCC3.27817).

Notes: In this study, two isolates corresponding to the genus *Bifusisporella* was collected from the *Bambusoideae*. The strain of the genus *Bifusisporella* was identified as a new species, with the two isolates representing one species. Phylogenetic analysis utilizing four genomic loci revealed that *B. magnum* constitutes a distinct clade, closely related to *B. bambooensis* (CGMCC3.25653), and this classification is strongly supported by statistical evidence (98% ML/1 pp). Compared to *B. bambooensis, B. magnum* sp. nov. has larger *conidiogenous cells* and smaller *conidia* (18.8–30.4 × 4.6–6.4 vs. 7.2–21.0 × 4.2–6.4 μm; 11.8–28.9 × 2.1–4.3 vs. 10.8–45.0 × 2.8–4.9 μm); Molecularly, an unnamed sequence that is at least 97% similar to a fully annotated type-derived sequence in a global ITS alignment can be annotated at the family level and often at the genus level (Nilsson et al., 2019), the nucleotide comparison of ITS, LSU, *tef1-*α, and *rpb1* (CGMCC3.27816) showed differences with the sequences of *B. bambooensis* (CGMCC3.25653), similarities are 16.1% (86/534), 2.8% (22/779), 3.7% (32/869), and 11.6% (73/628), therefore, the strain of the genus *Bifusisporella* was identified as a new species.

**Figure 4:**
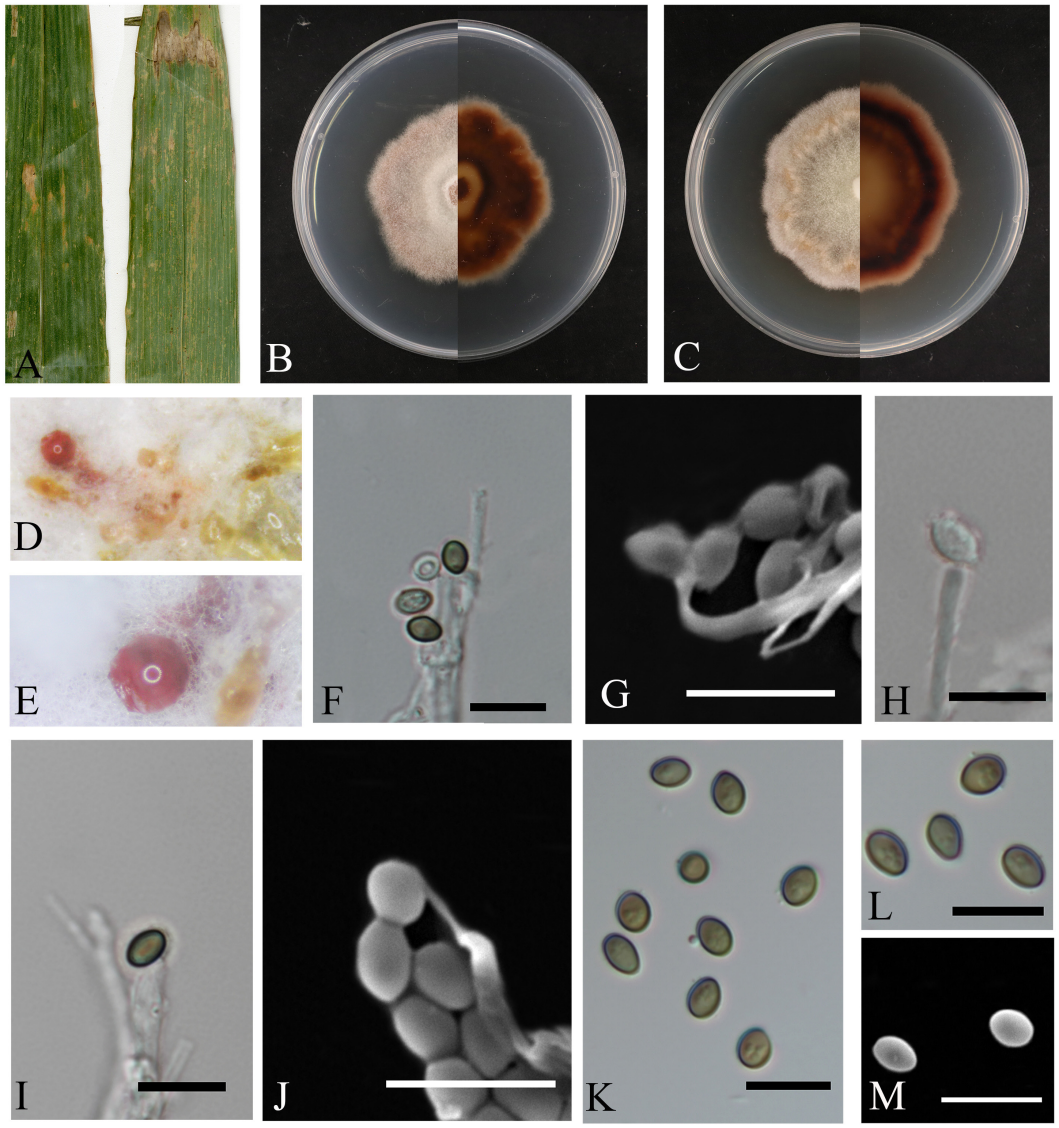
*Letendraea bambusae* (HMAS353152). **(A)** Leaves of host plant. **(B, C)** Upper and reverse view of colony after incubation for 7 days on PDA and 14 days. **(D, E)** Conidiomata sporulating on PDA. **(F–J)** Conidiogenous cells and conidia. **(K–M)** Conidia. **(F–M)** Scale bars: 10 μm.

***Letendraea bambusae*** sp. nov. Z.Y. Zhao, X.Y. Guan and J.Z. Qiu (Figure 4).

MycoBank: MB856575

Holotype: China, Fujian Province, Hua'an Bamboo Garden (117°32′23.35″E, 25°0′29.47″N), from diseased leaves of bamboo. June 2023, Z.Y. Zhao (holotype HMAS353152; ex-type living culture CGMCC3.28309).

Etymology: The epithet “bambusae” refers to the host, which is bamboo.

Description: The leaf spots are irregular, with colors transitioning from reddish-brown on the outer edges to white at the center. *Conidiomata* bulging on agar, transparent to pink, spherical in aggregate; *conidiophores* hyaline, smooth, subcylindrical, *conidia* are round or oval, with smooth surface, initially transparent and becoming olive or brown upon maturation, 3.6–5.9 × 2.1–3.9 μm, mean ± SD = 4.7 ± 0.6 × 3.0 ± 0.4 μm, n = 40.

Culture characteristics: the colonies on PDA are fluffy and velvety, with the colony surface appearing white, the reverse side of the colony transitions from a nearly circular, pale-yellow center to a reddish-brown color as it extends outwards. The growth rate was 0.7 cm/day.

Material examined: China, Fujian Province, Hua'an Bamboo Garden (117°32′23.35″E, 25°0′29.47″N), from diseased leaves of bamboo. June 2023, Z.Y. Zhao (paratype HMAS353153; ex-paratype living culture CGMCC3.28310).

Notes: In this study, the two isolates originated from diseased bamboo leaves. Phylogenetic analysis revealed that these isolates represent the same species and cluster together with *Letendraea* sp. (UTHSC: DI16-267) on a single clade, with high statistical support for their differences (BYPP = 1, ML-BS = 84%), the nucleotide comparison of ITS, LSU, SSU, and *tef1-*α (CGMCC3.28309) showed differences with the sequences of a closely related species *Letendraea helminthicola* (MFLUCC 19-0055), similarities were 19.0% (104/548), 57.5% (454/790), 1.0% (10/954), and 73.6% (559/759).

## 4 Discussion

The subtropical region of China is considered as an important area rich in biodiversity and a significant nature reserve for endemic plants (Dai et al., 2018). Fujian Province is located in the eastern part of the subtropical region, and rainfall comes from the East Asian monsoon, which determines the vegetation characteristics of East Asia (Webster et al., 1998). The main vegetation are evergreen broad-leaved forests, and the main tree species in Fujian include Masson's pine, bamboo forests, and willow trees (Zhu and Tan, 2024). The diversity of tree species in Fujian, combined with a warm and humid climate, is an important factor in promoting the proliferation of related fungi (Liu et al., 2024). In this study, two new species (*Bifusisporella magnum* sp. nov., and *Letendraea bambusae* sp. nov.) from Fujian Province, China are identified and described.

Members of the *Magnaporthaceae* family exists in all trophic levels of plants, including roots, stems, and leaves (Shearer et al., 1999). Many of these fungi display host preferences, especially those parasitic on the plants of *Poaceae* and *Cyperaceae* (Luo and Zhang, 2013; Yuan et al., 2010). In a study of endophytic fungi in Brazilian two-color sorghum (*Sorghum bicolor*) leaves, *Bifusisporella* was proposed as a new genus based on asexual morphological characteristics such as macroconidia and microconidia (Silva et al., 2019). Currently, four *Bifusisporella* species have been reported isolated healthy sorghum leaves (Silva et al., 2019). *B. sichuanensis* was proposed by Zeng et al. (2022) for strains isolated from the leaves of poplar trees in Sichuan Province, China, while *B. fujianensis* and *B. bambooensis* were proposed by Zhao et al. (2024) for strains isolated from diseased bamboo leaves. Here, *B. magnum* sp. nov. is also proposed for strains isolated from diseased bamboo leaves. It is likely that these fungi evolved from endophytic predecessors, as evidenced by conserved traits of appressorium development and lignocellulose-degrading enzyme secretion (Bhunjun et al., 2024). Many endophytic fungi within the *Magnaporthales* order possess the enzymatic machinery for cellulose and lignin decomposition, despite these functions being non-essential for their endophytic existence. The majority of *Bifusisporella* species generate melanized appressoria that closely resemble those observed in pathogenic fungi in both morphology and structure. Phylogenetic conservation of infection structures (appressoria) and lignocellulolytic machinery among *Magnaporthales* endophytes indicates evolutionary predisposition for niche flexibility, including possible pathogenic/saprobic conversions under ecological pressures (Konta et al., 2023; Feng et al., 2024).

Saccardo (1880) introduced the genus *Letendraea*, with *L. eurotioides* as its type species. Species of *Letendraea* often cause infections in various terrestrial habitats and have been associated with leaf spot diseases in the asparagus (*Cordyline* sp.) family. These fungi exhibit a similar morphology to *Wilmia*, a monotypic genus, containing the single species *Wilmia brasiliensis*. However, *Wilmia* (formerly known as *Phaeosphaeriaceae*) has been demonstrated to be synonymous with *Letendraea* (*Didymosphaeriaceae*) (Ariyawansa et al., 2014b). Currently, *Letendraea* contains four strains with *L. eurotioides* (CBS 212.31), *L. padouk* (CBS 485.70), *L. helminthicola* (CBS 884.85), *L. cordylinicola* (MFLUCC11-0148^T^, MFLUCC 11-0150). Our data add one new species to this group, *L. bambusae* sp. nov., along with a detailed description and its morphological illustration.

The taxonomic study of bambusicolous fungi has become an important research topic due to the increasing cultivation and use of bamboo worldwide (Dai et al., 2018; Phookamsak et al., 2022). East Asia, as the global center of bamboo distribution (especially China, Japan, and Korea), has rich bamboo species resources (Bambusoideae), but in-depth studies on the phylogenetic classification of bambusicolous fungi remain scarce (Hyde et al., 2002), resulting in a lack of molecular data that could help elucidate a significantly greater proportion of the diversity of bamboo associating fungi. Our data describe two new species and provides additional resources in the exploration of the ecology of bambusicolous fungi in East Asia. These results expand our knowledge of bambusicolous fungi, and also provides new insights into the range and potential specificity of their interactions in East Asia.

## Data Availability

The datasets presented in this study can be found in online repositories. The names of the repository/repositories and accession number(s) can be found in the article.
